# ∆^9^-Tetrahydrocannabinol, a major marijuana component, enhances the anesthetic effect of pentobarbital through the CB_1_ receptor

**DOI:** 10.1007/s11419-018-0457-2

**Published:** 2018-11-24

**Authors:** Toshiyuki Kimura, Makiko Takaya, Noriyuki Usami, Kazuhito Watanabe, Ikuo Yamamoto

**Affiliations:** 1 Department of Hygienic Chemistry, Faculty of Pharmaceutical Sciences, Hokuriku University, Ho-3, Kanagawa-machi, Kanazawa, 920-1181 Japan; 2 Center for Supporting Pharmaceutical Education, Daiichi University of Pharmacy, 22-1 Tamagawa-cho, Minami-ku, Fukuoka, 815-8511 Japan

**Keywords:** ∆^9^-Tetrahydrocannabinol, Cannabidiol, Pentobarbital-induced sleep, CB_1_ receptor, Cannabinoid

## Abstract

**Purpose:**

∆^9^**-**Tetrahydrocannabinol (∆^9^-THC) and cannabidiol (CBD), major psychoactive constituents of marijuana, induce potentiation of pentobarbital-induced sleep in mice. We have elucidated the mechanism of enhancement of the anesthetic effect of pentobarbital by cannabinoids.

**Methods:**

We carried out pharmacological experiment and cannabinoid_1_ (CB_1_) receptor binding assay using CB_1_ antagonists to clarify whether the CB_1_ receptor is involved in the synergism or not. The affinities of cannabinoids for the CB_1_ receptor in the mouse brain synaptic membrane were evaluated using a specific CB_1_ ligand, [^3^H]CP55940.

**Results:**

Although the potentiating effect of ∆^9^-THC on pentobarbital-induced sleep was attenuated by co-administration of CB_1_ receptor antagonists, such as SR141716A and AM251, at a dose of 2 mg/kg, intravenously (i.v.) to mice, the CBD-enhanced pentobarbital-induced sleep was not inhibited by SR141716A. The inhibitory constant (Ki) values of ∆^9^-THC and CBD were 6.62 and 2010 nM, respectively, showing a high affinity of ∆^9^-THC and a low affinity of CBD for the CB_1_ receptor, respectively. A high concentration of pentobarbital (1 mM) did not affect specific [^3^H]CP55940 binding on the mouse brain synaptic membrane.

**Conclusions:**

These results suggest that binding of ∆^9^-THC to the CB_1_ receptor is involved in the synergism with pentobarbital, and that potentiating effect of CBD with pentobarbital may differ from that of ∆^9^-THC. We successfully demonstrated that ∆^9^-THC enhanced the anesthetic effect of pentobarbital through the CB_1_ receptor.

## Introduction

While the current number of people arrested for marijuana (*Cannabis sativa L*)-related crimes under the Cannabis Control Law in Japan has decreased relatively over the past 10 years, there has been a gradually increase since 2014 [1]. A high proportion of people are arrested in their 20s, accounting for 42.4% of cannabis crimes, and a high ratio of first-time offenders is still observed. Despite prevention education of drug abuse being widespread, drug abuse-related crimes among young generations in Japan continue to exist. Moreover, cannabis continues to be the most widely cultivated, produced, trafficked and consumed drug worldwide, specifically in places such as North America, South America, Caribbean, and Africa, etc. Global number of users are estimated at 182.5 million [2]. Therefore, continuing scientific study of the interaction between drug toxicity and risk leads to the prevention of drug abuse.

Marijuana is positioned as a global gateway drug and its potential use with other abused drugs is a serious problem [3]. Furthermore, most cannabis components have oil-soluble properties [4], which upon entering the body, can become widely distributed due to these properties. In many cases, cannabis smokers continue to take other abused drugs, which may cause excess pharmacological interactions. Thus, it is important to understand the mechanism of drug-to-drug interactions when contributing to the prevention of drug abuse.

**Fig. 1:**
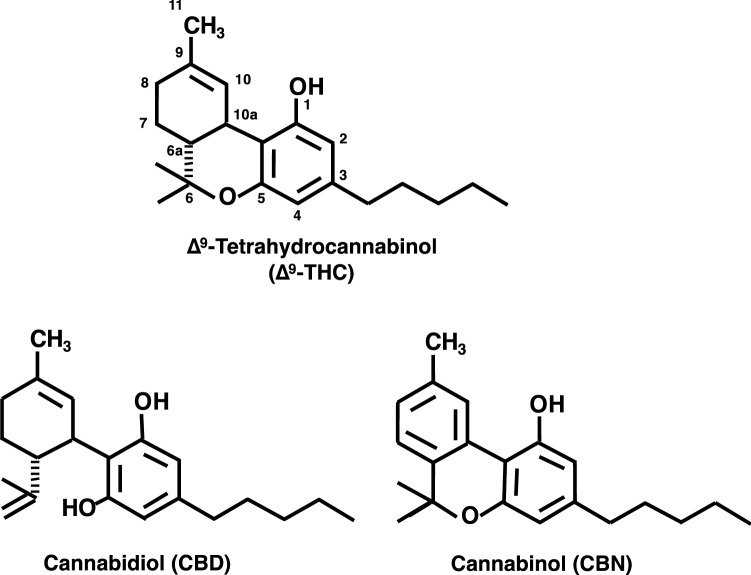
Chemical structures of major marijuana constituents

Marijuana contains a number of cannabinoids and the primary psychoactive component is ∆^9^-tetrahydrocannabinol (∆^9^-THC; Fig. 1). ∆^9^-THC is known to exhibit numerous pharmacological effects, such as catalepsy, hypothermia, analgesic effects, immobility, motor incoordination, and certain central nervous system (CNS) effects [5–7]. As shown in Fig. 1, the other major constituents of marijuana are cannabidiol (CBD) and cannabinol. CBD also shows certain pharmacological effects, such as anticonvulsant effects and synergism with CNS drugs, including barbiturates [8–10]. In terms of relative potencies of maximal electroshock test, CBD and ∆^9^-THC are similar, but both of them are more active than cannabinol [8]. CBD and ∆^9^-THC also prolonged pentobarbital-induced anesthesia [10].

Cannabinoid receptors are found in both the mammalian brain and peripheral organs, and are subdivided into central (CB_1_) and peripheral (CB_2_) receptors. Furthermore, the nucleotide sequences of both human receptors have been elucidated [11, 12]. The pharmacological effects of ∆^9^-THC on the CNS, such as hypoactivity, hypothermia, and antinociception, were reversed by the co-administration of the CB_1_ receptor antagonists SR141716A [*N*-(piperidin-1-yl)-5-(4-chlorophenyl)-1-(2,4-dichlorophenyl)-4-methyl-1H-pyrazole-3-carboxamide] and AM251 [*N*-(piperidin-1-yl)-5-(4-iodophenyl)-1-(2,4-dichlorophenyl)-4-methyl-1H-pyrazole-3-carboxamide] [13–15], indicating that these CNS effects were mediated by the CB_1_ receptor. Further evidence of CNS effects of cannabinoids on the CB_1_ receptor includes pharmacological effects and binding affinity of the brain CB_1_ receptor [16, 17]. We revealed that certain cannabinoids, their metabolites, and synthetic cannabinoids caused a pentobarbital-induced sleep-prolonging effect, in addition to catalepsy, hypothermia, and reduced spontaneous activity in mice [18–26]. Conversely, CBD is also known to prolong pentobarbital-induced sleep, but the potentiation mechanism was thought to differ to ∆^9^-THC. Namely, the CB_1_ receptor affinity of CBD is quite low and pentobarbital-induced sleep-prolonging effects with CBD were due to inhibition of hepatic cytochrome P450 activity [27–30]. Our previous data show that the pharmacological profile of each cannabinoid, including active metabolites such as 11-hydroxy-∆^9^-THC and 11-oxo-∆^9^-THC, based on the effects of hypothermia, catalepsy, and pentobarbital-induced sleep, differ widely in their site of action in the CNS [18–26]. However, the action mechanism of cannabinoids concerning synergism with certain anesthetics has not yet been clearly elucidated. In the present study, we reveal the involvement of the CB_1_ receptor for potentiating pentobarbital-induced sleep by ∆^9^-THC in mice using specific CB_1_ receptor antagonists.

## Materials and methods

### Drugs and reagents

[^3^H]CP55940 [(-)-*cis*-3-[2-hydroxy-4(1,1-dimethyl-heptyl)phenyl]-*trans*-4-(3-hydroxy- propyl)cyclohexanol; 112.8 Ci/mmol] was obtained from Daiichi Pure Chemical Co., Ltd./NEN (Tokyo, Japan). AM251 and sodium pentobarbital were purchased from Tocris Cookson Ltd. (Bristol, UK) and Tokyo Chemical Industry Co., Ltd., (Tokyo, Japan), respectively. Δ^9^-THC was isolated and purified from cannabis leaves according to the method of Aramaki et al. [31]. SR141716A was gifted from Sanofi-Synthelabo Recherche (Long Jumeau, France). Clearsol was obtained from Nacalai Tesque (Kyoto, Japan). All other chemicals were purchased from Wako Pure Chemicals, Ltd. (Osaka, Japan).

### Effect of cannabinoid receptor antagonists on cannabinoid-induced synergism with pentobarbital

For the pharmacological studies, cannabinoids and antagonists were suspended in saline containing 1% Tween 80. Sodium pentobarbital was dissolved in saline and injected intraperitoneally (i.p.; 40 mg/kg) for the mice. Male ddY mice weighing 20–25 g were housed in groups of 8. All animals were kept in a temperature-controlled (25 °C) environment with a 12-h light–dark cycle (lights on at 7:00 a.m.) and received food and water ad libitum. SR141716A or AM251 (2–10 mg/kg) was administered intravenously (i.v.) 10 min before i.v. injection with ∆^9^-THC 10 mg/kg. Sodium pentobarbital (40 mg/kg) was injected i.p. 15 min after the injection of ∆^9^-THC, after which sleeping time was measured as the time between loss of righting reflex and recovery. For CBD-induced pentobarbital potentiation, CBD 10 mg/kg was administered by i.v. injection to the mice instead of ∆^9^-THC. One percent Tween 80-saline solution was injected instead of cannabinoids or antagonists for the vehicle treated group as a control.

### Membrane preparation

Crude synaptic membranes from ddY male mice brains were prepared according to the method of Zukin et al. [32] with a slight modification. Briefly, whole mice brains were homogenized in 15 volumes of ice-cold 0.32 M sucrose for 1 min with a Polytron homogenizer (Kinematica, Lucerne, Switzerland), followed by centrifugation at 1000 g for 10 min at 4 °C. The pellet was discarded and the supernatant was centrifuged at 20000*g* for 20 min at 4 °C. This pellet was homogenized for 15 s in 40 volumes of ice-cold distilled water and further dispersed with a Polytron homogenizer. The suspension was then centrifuged at 8000*g* for 20 min at 4 °C. The supernatant and the soft buffy upper layer of the pellet were carefully collected and combined. This fraction was then centrifuged at 48000*g* for 20 min at 4 °C. The resulting pellet was homogenized for 15 s in 40 volumes of buffer A (50 mM Tris/HCl, 1 mM Tris/EDTA, 3 mM MgCl_2_; pH 7.4) at 37 °C. After centrifugation at 48000*g* for 20 min at 4 °C, the pellet was homogenized for 15 s in 10 volumes of the buffer A and stored at −80 °C for at least 24 h.

For the binding assay, the frozen membrane fraction was thawed and suspended in 40 volumes of buffer A. The suspension was incubated at 37 °C for 30 min following centrifugation at 48000*g* for 20 min at 4 °C. The pellet was then homogenized in enough buffer A to give a protein concentration of approximately 0.25 mg/ml. Protein determination was performed according to the method of Lowry et al. [33].

### Cannabinoid receptor binding assay

A ligand binding assay for the cannabinoid receptor was performed according to the method of Devane et al. [34] and Compton et al. [16]. Incubations were done in siliconized glass tubes, each containing 0.15 mg of synaptic membrane protein from mice brains, 0.2 nM [^3^H]CP55940, 5 mg of fatty acid-free bovine serum albumin (BSA), and various concentrations of cannabinoid receptor ligands in 1 ml of buffer A. The mixture was incubated at 37 °C for 1 h. Whatman GF/B filters were allowed to stand in buffer A containing 0.1% polyethyleneimine for at least 1 h before the assay. The incubation was terminated by filtration on Whatman GF/B filters using a cell harvester (Brandel model M-24) and filters were then washed twice with 5 ml of cold buffer B (50 mM Tris/HCl, 1 mM Tris/EDTA, 3 mM MgCl_2_, and 1 mg/ml BSA; pH 7.4 at 30 °C). The filters were then placed in glass scintillation vials with 10 ml of Clearsol and the radioactivity was counted using a liquid scintillation counter (LSC-6100, Aloka, Tokyo, Japan). Nonspecific binding obtained in the presence of 10 µM of Δ^9^-THC was subtracted from total binding to determine specific binding. The displacement curve of specific [^3^H]CP55940 binding was fitted by ORIGIN ver. 7.5 (OriginLab, MA, USA), after which we calculated the 50% inhibitory concentration (IC_50_). The inhibitory constant (Ki value) was calculated according to the method of Speth et al. [35].

### Statistical analysis

Statistical evaluation of the data was performed by using the Bonferroni analysis of variance [36].

## Results and discussion

**Fig. 2:**
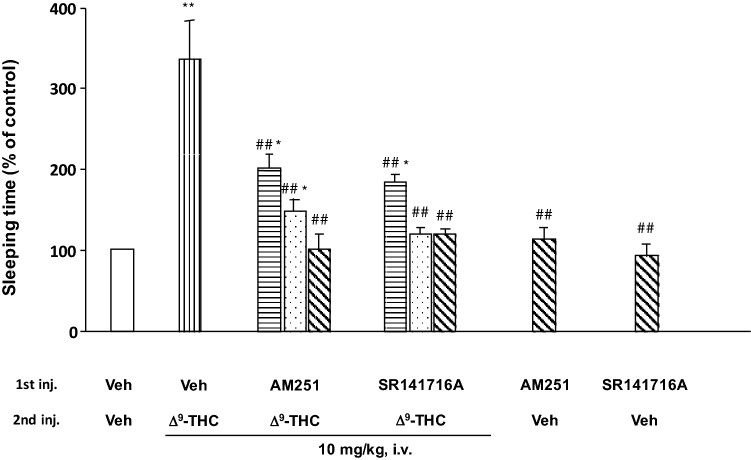
Effects of CB_1_ receptor antagonists on ∆^9^-THC-induced pentobarbital potentiation in mice. Mice were pretreated with 2, 5, or 10 mg/kg of CB_1_ receptor antagonists by i.v. injection 10 min before ∆^9^-THC administration (10 mg/kg, i.v.), and then administered 40 mg/kg of pentobarbital by i.p. injection (*n* = 8). Sleeping time was expressed as the mean % of control (Veh + Veh + pentobarbital) sleeping time. Concentration of CB_1_ receptor antagonist:  2 mg/kg, i.v.,  5 mg/kg, i.v.,  10 mg/kg, i.v., Control sleeping time was 37 ± 5 min. Significant difference from control group (Veh + Veh; *p < 0.05, **p < 0.01). Significant difference from ∆^9^-THC alone treated group (Veh + ∆^9^-THC; ##*p* < 0.01)

The effects of cannabinoid receptor antagonists on the synergistic effects of pentobarbital with ∆^9^-THC are shown in Fig. 2. ∆^9^-THC [vehicle (Veh) + ∆^9^-THC 10 mg/kg, i.v.] significantly prolonged pentobarbital-induced sleep by 3.3-folds compared with the vehicle-pretreated group (Veh + Veh). The result supports our previous finding that ∆^9^-THC prolonged pentobarbital-induced sleeping time in mice by various routes of administrations [22]. At this time, once an abused drug, such as pentobarbital, has entered the body, a drug interaction between ∆^9^-THC and pentobarbital may occur. Moreover, this should be treated as a serious problem, rather than simply a case of single administration of cannabis or barbiturates alone.

The cannabinoid receptor is subdivided into CB_1_ and CB_2_ receptors, with each specific ligand (agonist and antagonist) previously determined [37]. In the present study, SR141716A and AM251 were used as CB_1_ receptor antagonists to investigate pharmacological effects and perform the receptor binding affinity experiment. Compton et al. [14] reported antagonistic response of SR141716A for pharmacological and behavioral experiments such as measurements of spontaneous locomotor activity, tail-flick responsiveness, or rectal temperature at the range of 1–10 mg/kg, i.v. to mice. We also used an equivalent dose of SR141716A for the effect of ∆^9^-THC-induced pentobarbital potentiation. When SR141716A or AM251 (2–10 mg/kg) was pre-administered by i.v. injection 10 min before ∆^9^-THC treatment, the potentiation of pentobarbital-induced sleep by ∆^9^-THC decreased dose-dependently. In particular, a low dose of the CB_1_ receptor antagonists (2 mg/kg, i.v.) significantly suppressed the prolonging effect of ∆^9^-THC. Cannabinoid receptor antagonists themselves (AM251 + Veh or SR141716A + Veh) then caused no change in pentobarbital-induced sleeping time. The attenuated synergistic effect of ∆^9^-THC on pentobarbital-induced sleep was thought to be due to blockade of CB_1_ receptor binding by specific CB_1_ receptor antagonists.

**Fig. 3:**
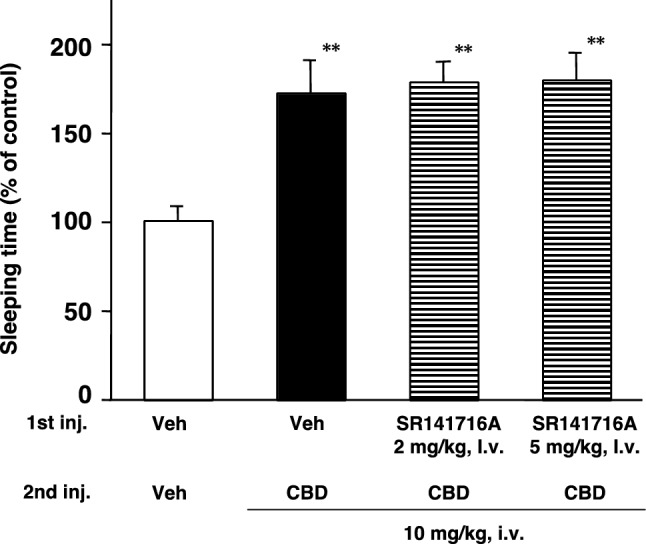
Effects of CB_1_ receptor antagonist on CBD-induced pentobarbital potentiation in mice. Mice were pretreated with 2 or 5 mg/kg SR141716A by i.v. injection 10 min before CBD administration (10 mg/kg, i.v.) and then administered pentobarbital at 40 mg/kg by i.p. injection. Sleeping time was expressed as the mean % of control (Veh + Veh + pentobarbital) sleeping time (*n* = 8). Control sleeping time was 35 ± 3 min. Significant difference from control group (**p < 0.01)

Since a low dose of CB_1_ receptor antagonists attenuated the synergistic effects of pentobarbital with Δ^9^-THC and CB_1_ receptor affinity of SR141716A is much higher than the affinity of AM251, doses of 2 or 5 mg/kg, i.v. of SR141716A were used for the synergistic experiment of CBD and pentobarbital. CBD (Veh + CBD 10 mg/kg, i.v.) also significantly prolonged pentobarbital-induced sleep 1.8-fold compared with the control (Veh + Veh), while pretreatment with SR141716A (2 or 5 mg/kg, i.v.) failed to inhibit CBD-enhanced pentobarbital-induced sleep (Fig. 3). CBD is well known as a major constituent of marijuana, causing pharmacological effects, such as barbiturate synergism [8–10, 24]. Watanabe et al. [30] previously reported the potentiation mechanism of CBD on pentobarbital-induced sleep, which is caused by inhibition of the hepatic drug-metabolizing enzymes by CBD. CBD inhibits the metabolism of pentobarbital, leading to increased levels of the drug in the body and enhances its action. Namely, the synergistic effect of CBD with pentobarbital is different from ∆^9^-THC, which reflects how the CB_1_ receptor influences CNS pharmacological effects. Therefore, this failure of SR141716A to produce antagonistic effects on CBD-induced pentobarbital potentiation suggests that CBD does not bind to the CB_1_ receptor in CNS at the supplied dosage of 10 mg/kg i.p. of CBD in mice.

**Fig. 4:**
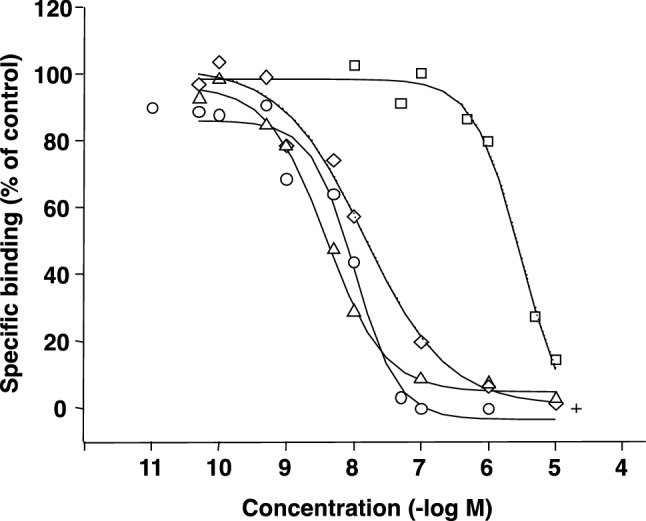
Displacement effects of ∆^9^-THC (circle), CBD (square), AM251 (triangle), and SR141716A (diamond) on specific [^3^H]CP55940 binding to the synaptic membrane from ddY mice brains. Displacement curves were fitted by ORIGIN ver. 7.5 and calculated as IC_50_

As shown in Fig. 4, the affinities of antagonists for CB_1_ receptor binding were evaluated using a radio receptor assay measuring specific [^3^H]CP55940 binding to the mouse brain synaptic membrane. All compounds concentration-dependently inhibited the specific [^3^H]CP55940 binding. ∆^9^-THC and CB_1_ receptor antagonists, except for CBD, possessed the same potency for the CB_1_ receptor binding. The Ki values of cannabinoid ligands are summarized in Table 1. The Ki values of ∆^9^-THC and CBD were 6.62 and 2010 nM, respectively, showing the high affinity of ∆^9^-THC and the low affinity of CBD for the CB_1_ receptor. The Ki values of CB_1_ receptor antagonists, SR141716A and AM251, were 9.54 and 2.58 nM, respectively, indicating the high affinities of these antagonists to the CB_1_ receptor binding site, as well as ∆^9^-THC. Yamamoto et al. [38] also reported that active metabolites of ∆^9^-THC bound to the cannabinoid CB_1_ receptor in the brain. The results confirm that the potentiation mechanism of pentobarbital-induced sleep by CBD is not mediated through the CB_1_ receptor, but rather by other mechanisms, such as inhibition of barbiturate metabolism (Table 2).

**Table 1:** Displacement of specific [^3^H]CP-55940 binding to the synaptic membrane from ddY mice brains by cannabinoid receptor ligands

Compound	IC_50_ (nM)	Ki (nM)
Major marijuana constituents		
∆^9^-THC	10.2	6.62
CBD	3100	2010
CB_1_ Antagonists		
SR141716A	14.7	9.54
AM251	3.98	2.58

*∆*^*9*^*-THC* ∆^9^-tetrahydrocannabinol, *CBD* cannabidiol

The involvement of pentobarbital in CB_1_ receptor function was also examined. We added 1 mM pentobarbital to the CB_1_ receptor binding assay mixture. However, pentobarbital did not affect specific [^3^H]CP55940 binding to the mice brain synaptic membrane (Table 2). In other words, pentobarbital did not directly affect the CB_1_ receptor binding site. The cannabinoid CB_1_ receptor antagonists, SR141716A and AM251, are effective in antagonizing in vivo effects of ∆^9^-THC, such as hypothermia and antinociception, as well as decreasing locomotor activity [14, 37]. To date, several lines of evidence indicate that cannabinoids potentiate barbiturate potency as a pharmacological property of marijuana [5, 7]. We also revealed that marijuana constituents, their active metabolites, and synthetic analogues prolonged pentobarbital-induced sleep in mice [18, 23, 24]. In the structure–activity relationship of cannabinoids showing CNS pharmacological effects, the cannabinoid-induced barbiturate synergism was similar to other CNS indices, such as catalepsy and hypothermia [21], suggesting a close interaction between cannabinoid and barbiturate action sites.

**Table 2:** Effect of pentobarbital on specific [^3^H]CP55940 binding to the synaptic membrane from mice brains

Compound	Specific [^3^H]CP55940 binding (pmol/mg protein)
Control	0.055 ± 0.002
Pentobarbital 1 mM	0.050 ± 0.003 ^N.S.^

Pentobarbital 1 mM was added to the reaction mixture

*NS* No significant difference from control binding

Many reports explore the barbiturate active site, but a precise action mechanism of barbiturates is not fully elucidated. In general, barbiturates act on the GABA_A_ supramolecule receptor complex, which couples with the GABA and benzodiazepine binding sites, as well as the picrotoxinin and chloride ion channel [39]. We previously reported the effects of cannabinoids on the benzodiazepine receptor of the synaptic membrane in the bovine brain [40]. ∆^9^-THC and its metabolites competitively bound to the benzodiazepine receptor, indicating a slight co-modification of benzodiazepine receptor binding by these cannabinoids. Despite this, the receptor binding affinities of the cannabinoids to the benzodiazepine receptor were low. For the synergy of barbiturate with ∆^9^-THC, the direct interaction of pentobarbital with the CB_1_ receptor was ruled out, since adding high concentrations of pentobarbital (1 mM) to the receptor binding mixture did not change the specific [^3^H]CP55940 binding to the membrane. In addition, treatment of CB_1_ receptor antagonists without Δ^9^-THC resulted in no change in sleeping time compared with vehicle treatment, indicating pentobarbital did not compete with the CB_1_ receptor binding site. Regarding the mechanism explaining the synergy between cannabinoids and barbiturate, the barbiturate action site differed from the CB_1_ receptor, suggesting the CB_1_ receptor signal may activate the barbiturate site downstream to the CB_1_ receptor site. CB_1_ receptors are known to be coupled through G protein-dependent and G protein-independent (Gi/o) receptors to certain ion channels [41]. The present results suggest that positive cross-talk is present between CB_1_ receptor signaling and pentobarbital-induced chloride channels. However, concerning the action mechanism downstream of the CB_1_ receptor binding site is still unknown. It may be related to a certain signal transduction between the cannabinoid receptor and barbiturate active sites, including the GABA_A_ receptor and G protein. Since the CB_1_ receptor is known to couple Gi/o protein, measurement of adenylate cyclase activity of CB_1_ receptor in the presence of pentobarbital may be evidence of this interaction. Moreover, barbiturates are known to affect to GABA_A_ receptor. Study of involvement of GABA_A_ receptor to CB_1_ receptor is future work.

Our study suggested that the enhanced anesthetic effect of pentobarbital with ∆^9^-THC results in an interaction between the CB_1_ receptor and barbiturate action site.

## Conclusions

∆^9^-THC induced pentobarbital potentiation in mice and this potentiation was blocked by specific CB_1_ receptor antagonists, SR141716A and AM251. The affinities of CB_1_ receptor antagonists to the mouse brain synaptic membrane were similar to blockage effects on pentobarbital-induced sleep potentiation by ∆^9^-THC. Pentobarbital did not directly affect specific CB_1_ receptor binding, suggesting that ∆^9^-THC and barbiturate did not share the same active site. Since CB_1_ receptor antagonists blocked ∆^9^-THC potentiating pentobarbital-induced sleep, the interaction site of pentobarbital may be located downstream of the CB_1_ receptor. The pharmacological results indicate the effect of ∆^9^-THC co-administered with pentobarbital was a synergistic, but not additive, action in mice. Further evidence suggests the CB_1_ receptor plays an important role as a trigger in potentiating pentobarbital-induced sleep by ∆^9^-THC.
